# Evaluation of homologous recombination testing in ovarian carcinoma

**DOI:** 10.1007/s00428-026-04432-2

**Published:** 2026-02-07

**Authors:** Vera M. Witjes, Joanne A. de Hullu, Angela van Remortele, Lilian Vreede, Efraim H. Rosenberg, Saskia A. G. M. Cillessen, Floris H. Groenendijk, Elisabeth M. P. Steeghs, Laura Moonen, Arjen R. Mensenkamp, Arja ter Elst, Wendy W. J. de Leng, Nicoline Hoogerbrugge, Marjolijn J. L. Ligtenberg

**Affiliations:** 1 Department of Human Genetics, Radboud University Medical Center, Nijmegen, the Netherlands; 2 Research Institute for Medical Innovation, Radboud University Medical Center, Nijmegen, the Netherlands; 3 Department of Obstetrics and Gynecology, Radboud University Medical Center, Nijmegen, the Netherlands; 4 Department of Pathology, Netherlands Cancer Institute, Amsterdam, the Netherlands; 5 Department of Pathology, Amsterdam University Medical Center, Amsterdam, the Netherlands; 6 Department of Pathology and Clinical Bioinformatics, Erasmus MC Cancer Institute, University Medical Center Rotterdam, Rotterdam, the Netherlands; 7 Department of Pathology, Leiden University Medical Center, Leiden, the Netherlands; 8 Department of Pathology, GROW School for Oncology and Reproduction, Maastricht University Medical Centre, Maastricht, the Netherlands; 9 Department of Pathology, Radboud University Medical Center, Nijmegen, the Netherlands; 10 Department of Pathology and Medical Biology, University of Groningen, University Medical Center Groningen, Groningen, the Netherlands; 11 Department of Pathology, University Medical Center Utrecht, Utrecht, the Netherlands

**Keywords:** Homologous recombination deficiency, Ovarian cancer, PARP inhibitors, Genomic instability, BRCA, Quality control

## Abstract

Homologous recombination deficiency (HRD) testing may be used to stratify ovarian carcinoma (OC) patients for PARP inhibitor therapy. In the Netherlands, different NGS-based assays are used to assess genomic instability as a hallmark of HRD. We evaluated the uniformity of HRD testing. Firstly, interlaboratory assessments of 10 tumors were performed in 8 centers. 71 out of the 77 (92%) successful tests were concordant. Results were more consistent in OC with a pathogenic variation (PV) or promoter methylation of a homologous recombination repair (HRR) gene (97%) than in those without (87%). Secondly, concordance between *BRCA1*/*RAD51C* promoter methylation and HRD was assessed in 244 samples without a PV in HRR genes. *BRCA1*/*RAD51C* promoter methylation was present in 38 out of 100 (38%) samples classified as HRD, and absent in all (*n* = 144) non-HRD samples (*p* < 0.001). Lastly, pathology reports from 765 HRD tests were reviewed to evaluate routine diagnostics. Testing was successful in 695 (91%) cases. HRD detection rates were higher in high-grade serous OC compared to other histological subtypes (49% versus 12%, *p* < 0.001). The five HRD assays varied significantly in HRD detection rates in high-grade serous OC. The results support the applicability of genomic instability analyses to assess HRD, while also highlighting the need to improve harmonization across different assays when HRD is used for therapeutic decision making.

**Supplementary Information:**

The online version contains supplementary material available at 10.1007/s00428-026-04432-2.

## Introduction

Ovarian carcinoma (OC, including fallopian tube carcinoma and primary peritoneal carcinoma) is marked by late-stage at diagnosis and poor survival rates. The five-year survival is estimated to be 34% [1]. The recent introduction of Poly (ADP-ribose) polymerase (PARP) inhibitors into first-line maintenance treatment for women with advanced-stage OC has improved clinical outcomes, enhancing both progression-free and overall survival [2, 3]. PARP inhibitors demonstrate the highest magnitude of clinical benefit in patients with a germline or somatic *BRCA1/2* pathogenic variant (PV). Additionally, this class of drugs also shows increased effectiveness in *BRCA1/2* wild-type tumors with homologous recombination deficiency (HRD) [4].

The European Medicines Agency (EMA) approvals for the PARP inhibitor treatment in the OC first-line maintenance setting vary, ranging from exclusively in patients with a tumor *BRCA1/2* PV (e.g., olaparib), to broader use in the entire population (e.g., niraparib), or in combination with antiangiogenic therapy specifically for HRD-tumors (e.g., olaparib) [5, 6]. A European expert consensus stated that, to extend effective PARP inhibitor treatment to the largest number of patients, *BRCA1/2* tumor DNA testing should be accompanied by evaluating the HRD status of the tumor [7]. In the Netherlands, HRD testing was obligatory between January 2024 and June 2025 to stratify patients with OC for first-line maintenance treatment with niraparib [8–10].

The implementation of HRD testing in routine clinical practice is an ongoing challenge. The Association of Molecular Pathology recognizes these challenges and has recently published joint consensus recommendations to provide guidance on the detection of HRD [11]. In clinical trials, the MyChoice CDx test is used to identify HRD by testing presence of *BRCA1/2* PV and genomic instability [3, 12]. Therefore, this test has been considered the “best comparator” and Dutch laboratories validated and implemented orthological techniques that also measure genomic instability as a marker for HRD. These tests were implemented as an addition to the standard tumor DNA test that assesses the presence of the OC risk-associated homologous recombination repair (HRR) genes *BRCA1*, *BRCA2*, *RAD51C*, *RAD51D*, *BRIP1* and *PALB2* PV to inform on PARP inhibitor eligibility and to stratify germline testing (i.e., the Tumor-First workflow) [13]. The choice of assays varied across the centers.

Given the variation in HRD assays and limited understanding of the optimal measurement of genomic instability, quality control was crucial. This study aimed to evaluate the uniformity of HRD tests implemented in daily diagnostic practice in the Netherlands.

## Methods

This study aimed to evaluate the uniformity of HRD testing by (1) organizing interlaboratory quality evaluation rounds, (2) assessing the concordance between the presence of *BRCA1*/*RAD51C* promoter methylation status and the presence of HRD, and (3) monitoring the HRD testing results in daily diagnostic practice. This evaluation was not subject to the Medical Research Involving Human Subjects Act (WMO), as assessed by the local medical ethics committee of the Radboudumc (Number 2024–17668).

### Study setting

In the Netherlands, HRD testing was implemented in the seven university medical centers and the Netherlands Cancer Institute between January 2024 and January 2025. Five centers implemented the TruSight Oncology 500 HRD assay (TSO 500), while the remaining three centers each adopted a different technique: whole-exome sequencing with PureCN algorithm (WES) [14], the Oncomine Comprehensive Assay Plus (OCA Plus) [15, 16], and shallow whole genome sequencing with the *BRCA1/2* classifier score (sWGS) [17], respectively. Both TSO 500 and WES calculate a genomic instability score (GIS) and use a threshold of 42, while the OCA Plus calculates a genomic instability metric (GIM) with a threshold of 16, and the *BRCA1/2* classifier score has a threshold of 0.5. Tumors with scores ≥ the threshold are assessed as HRD. Besides these techniques that assess presence of HRD on formalin-fixed paraffin-embedded (FFPE) tissue, one center also implemented whole genome sequencing (WGS) on fresh frozen tissue. WGS calculates a Classifier of Homologous Recombination Deficiency (CHORD) score and uses a threshold of 0.5.

### Interlaboratory quality rounds

Two interlaboratory quality rounds were organized to assess the concordance of HRD testing results. In both rounds, 5 ovarian tumors were selected from the molecular diagnostic database, either from Radboud university medical center or the Netherlands Cancer Institute. Tumors were selected based on the availability of material, the percentage of tumor cells, and variation in the presence of variants in or promoter methylation of HRR genes and variation in HRD scoring. For each case, participating laboratories received two unstained slides and one Hematoxylin and Eosin (H&E) stained slide from FFPE tissue. The laboratories were requested to analyze these samples using the standard diagnostic tumor testing protocol for OC (i.e., Tumor-First PV analysis and HRD analysis). Test reports were prepared in the regular format and returned for analysis. The results of the HRD analysis were compared, and the laboratories received feedback on these results after each round.

### Promoter methylation status

The *BRCA1*, *BRCA2*, and *RAD51C* promoter methylation status of tumors assessed as HRD and non-HRD was analyzed. DNA samples of OC without a PV in one of the evaluated HRR genes were requested from the eight testing laboratories in the Netherlands. Per laboratory, we requested 15 DNA samples of tumors scored as non-HRD and 15 samples scored as HRD. Since not all laboratories could provide these numbers, three centers were allowed to supply more, up to 50 cases. Within Radboud university medical center, a methylation-specific multiplex ligation-dependent probe amplification (MS-MLPA) was performed centrally following the manufacturer’s instructions (SALSA MLPA Probemix ME053 BRCA1-BRCA2-RAD51C, MRC-Holland, Amsterdam, the Netherlands). Samples were assessed as methylated when all probes were positive. The concordance of the promoter methylation status with the scored HRD status was analyzed, as well as the genomic instability measures of tumors with and without methylation.

### HRD testing results in clinical practice

To monitor HRD testing in clinical practice, patients newly diagnosed with OC, including epithelial ovarian cancer, fallopian tube carcinoma and primary peritoneal carcinoma, in 2023 or 2024, were obtained from the Netherlands Cancer Registry (NCR). Registration of patients diagnosed in 2024 in the cancer registry was incomplete due to the timing of data collection, which preceded full data entry into the registry. Therefore, the initial patient selection was extended by a search in the Dutch nationwide pathology databank (Palga) [18] for patients diagnosed with OC in 2024 (Supplementary Fig. 1). Of the selected patients, pathology reports were collected including updates until April 2025. Tumor characteristic data (e.g., histology) was obtained from the NCR, and HRD testing characteristics and results as well as Tumor-First and MSI testing information were obtained from the pathology reports. Scores ≥ the threshold were considered HRD. Outcomes included the overall testing characteristics and results for the total population, along with differences among the HRD assays.

### Data analysis

Descriptive statistics were reported as median and interquartile range (IQR) for continuous variables, and number (*n*) and percentage for categorical variables. Mann–Whitney U tests were used to assess differences in continuous variables, and Chi-square tests and Fisher exact tests were used for categorical variables. Pairwise comparisons of HRD detection rates across multiple categories were performed using estimated marginal means from logistic regression, with Bonferroni correction. Receiver operating characteristics (ROC) curves were constructed for the assay scores to predict the presence of a *BRCA1/2* PV. Optimal cut-offs were determined using the Youden index and 95% sensitivity.

Analyses were performed in R (v4.4.1) using RStudio (2024.04). Two-sided *p*-values < 0.05 were considered statistically significant.

## Results

### Interlaboratory quality rounds

All laboratories (*n* = 8) participated with their HRD tests used in daily diagnostics on FFPE material: TSO 500 (*n* = 5), OCA Plus (*n* = 1), WES (*n* = 1), and sWGS (*n* = 1). The results of the HRD quality rounds are presented in Table 1. The majority of the tests, 71 out of the 77 successful tests (92%) led to the same HRD status, while 6 (8%) of the 77 test results were discordant. The TSO 500 tests analyzed in 5 different centers yielded consistent results, while the other test types occasionally (WES and OCA Plus) or regularly (sWGS) deviated from those results. The test results of the samples with a known cause for HRD (PV in *BRCA1*/*RAD51C* or *BRCA1*/*RAD51C* promoter methylation) were more consistent (38/39 tests, 97% consistency) compared to the samples without a known cause for HRD (33/38 tests, 87% consistency). Of interest, 3 inconsistent test results were within two points of the threshold value of the test.

**Table 1:**
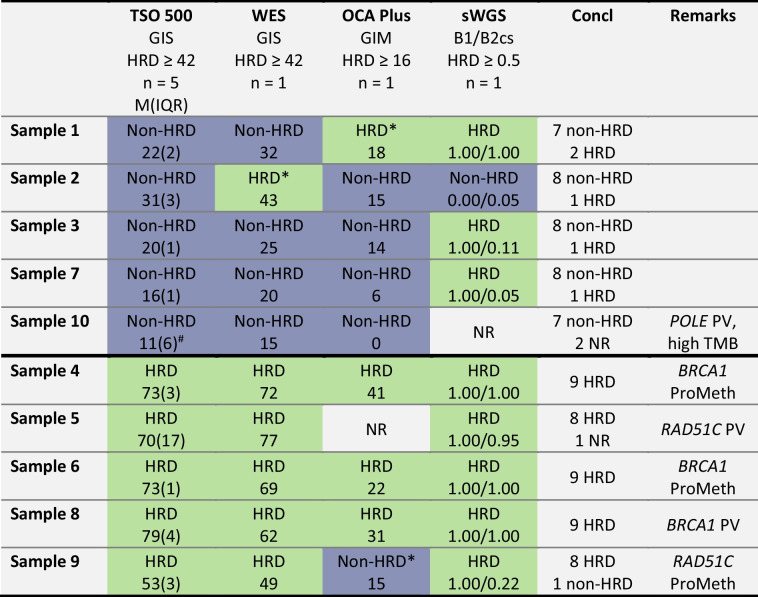
Summary of the results of the HRD testing quality rounds with ten formalin-fixed paraffin-embedded samples and four testing methods. The top five samples had no cause for homologous recombination deficiency, whereas the bottom five did have a pathogenic variant in or promoter methylation of *BRCA1* or *RAD51C*. Samples classified as non-HRD are displayed in purple, and those classified as HRD are in green

*Score just above/below (< 2) the threshold value of the test

#One of the five TSO 500 tests had no result

Abbreviations: TSO 500, TruSight Oncology 500 HRD test; WES, whole exome sequencing with PureCN algorithm; OCA Plus, Oncomine Comprehensive Assay Plus; sWGS, shallow whole genome sequencing with *BRCA1/2* classifier score; WGS, whole genome sequencing, GIS genomic instability score; GIM, genomic instability metric; B1/B2 cs, *BRCA1/BRCA2* classifier score; HRD, homologous recombination deficient; M(IQR), median(interquartile range); NR, no result; PV, pathogenic variant; ProMeth, promoter hypermethylation

### Promoter methylation status

**Fig. 1:**
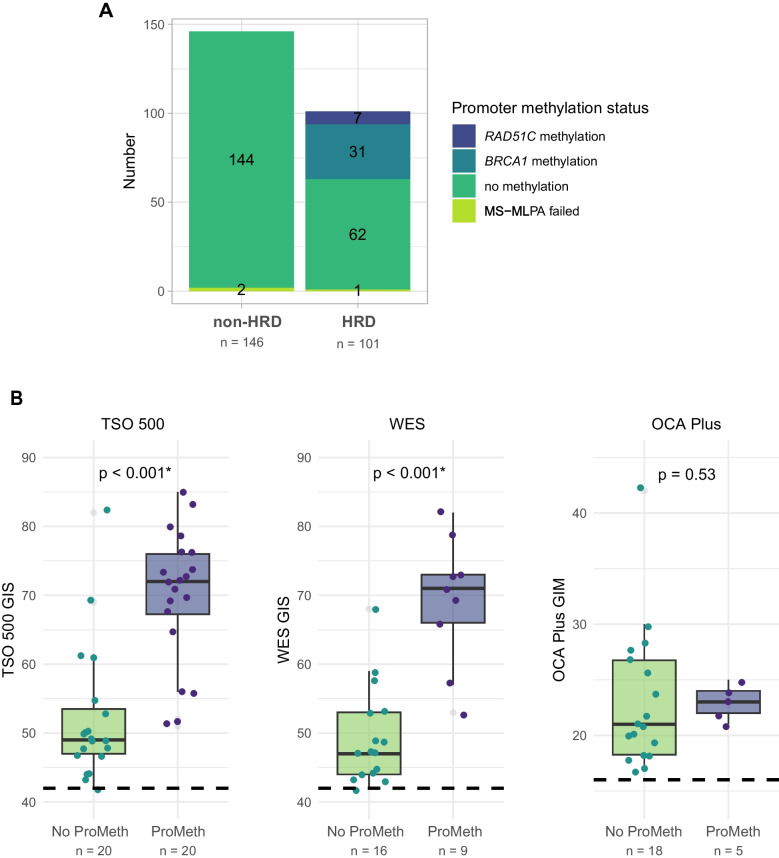
**A** Stacked bar chart showing promoter methylation status of samples assessed as non-HRD and HRD **B** Boxplots with overlaid scatter points showing GIS and GIM scores in HRD-positive tumors with and without promoter methylation, across TSO 500, WES, and OCA Plus tests. *P*-values are calculated with a Mann–Whitney *U* test. Asterisk (*) indicates a significant difference. Abbreviations: MS-MLPA, methylation-specific multiplex ligation-dependent probe amplification; TSO 500, TruSight Oncology 500 HRD test; WES, whole exome sequencing with PureCN algorithm; OCA Plus, Oncomine Comprehensive Assay Plus; GIS, genomic instability score; GIM, genomic instability metric; ProMeth, promoter methylation

Methylation of HRR genes was evaluated as it is expected to be restricted to cases with HRD and thus can serve as an indicator for false-negative HRD assay results. To this end, a total of 247 DNA samples without a PV detected in one of the HRR genes were collected for promoter methylation testing. HRD testing was performed locally using standard diagnostic methods. Most tumors (*n* = 123, 50%) were tested with the TSO 500, *n* = 50 were tested using WES (20%), *n* = 48 using OCA Plus (19%), *n* = 25 using sWGS (10%), and *n* = 1 using WGS. 146 tumors (59%) were assessed as non-HRD, and 101 (41%) were assessed as HRD, with scores representative of those observed in routine diagnostics. Figure  1A presents the results of the promoter methylation test. The promoter methylation test was successful in 144 out of the 146 samples scored as non-HRD and in none of these samples was promoter methylation detected. Out of the 100 samples scored as HRD that were successfully tested, *n* = 31 and *n* = 7 were found to have *BRCA1* and *RAD51C* promoter methylation, respectively. Thereby, promoter methylation was significantly more frequent in the HRD group in comparison to the non-HRD group (38% compared to 0%, Fisher’s exact test *p* < 0.001). Within the group assessed as HRD, the genomic instability score (GIS) determined by TSO 500 and WES was significantly higher in the samples with promoter methylation than in the samples without promoter methylation (Mann–Whitney U test, *p *< 0.001 for both assays), as presented in Fig. 1B. No significant difference in Genomic Instability Metric (GIM) was observed for the samples tested with OCA Plus. For the other test types, such a comparison was not possible given the available parameters.

### HRD testing results in clinical practice

#### HRD testing characteristics and results

Pathology reports from 2331 patients diagnosed with OC in 2023 or 2024 were reviewed. HRD testing, implemented in 2024, was performed in 765 of these patients (Supplementary Fig. 1). The general characteristics of these HRD tests are presented in Table 2. The median age of the patients was 67 years, and 61% had high-grade serous OC (HGSOC). In the tested population, the distribution of histology differed slightly but significantly from that of the total OC population (χ^2^ test, *p *< 0.001), with a higher proportion of HGSOC (Supplementary Table 1). The majority of HRD tests, 59%, were conducted on resected tumor tissue, while biopsies and cytology specimens accounted for 32% and 9%, respectively. In total, 695 of the 765 HRD tests (91%) were performed successfully, and HRD was identified in 241 (35%) of the successful tests. HRD was detected more frequently amongst the HGSOC compared to the group of other histological subtypes (49% compared to 12%, χ^2^ test, *p* < 0.001), as visualized in Supplementary Fig. 2A.

**Table 2:** General characteristics of the HRD tests in clinical diagnostics

**Patient age** (***N*** = 765)	Median (IQR)	67 (57–74)
**Year of diagnosis** (***N*** = 765)	2024	636 (83.1%)
	2023	129 (16.9%)
**Histology** (***N*** = 765)	High-grade serous	465 (60.8%)
	Clear cell	57 (7.5%)
	Mucinous	52 (6.8%)
	Endometrioid	45 (5.9%)
	Low-grade serous	45 (5.9%)
	(Adeno)carcinoma NOS	39 (5.1%)
	Serous NOS	31 (4.1%)
	Carcinosarcoma	21 (2.7%)
	Other	10 (1.3%)
**Tumor material** (***N*** = 765)*	Resection	449 (58.7%)
	Biopsy	245 (32.0%)
	Cytology	70 (9.2%)
	Unknown	1 (0.1%)
**Type of HRD test** (***N*** = 765)*	TSO 500	307 (40.1%)
	WES	241 (31.5%)
	OCA Plus	106 (13.9%)
	sWGS	62 (8.1%)
	WGS	49 (6.4%)
**HRD test successful** (***N*** = 765)	Successful in one test	678 (88.6%)
	Not successful in one test	60 (7.8%)
	Successful in two tests	17 (2.2%)
	Not successful in two tests	10 (1.3%)
**HRD test result** (***N*** = 695)	Non-HRD	454 (65.3%)
	HRD	241 (34.7%)

*For the patients that received an additional test because the first test was not successful, the information from the last test is used

HRD, homologous recombination deficiency; IQR, interquartile range; NOS, not otherwise specified; TSO 500, TruSight Oncology 500 HRD test; WES, whole exome sequencing with PureCN algorithm; OCA Plus, Oncomine Comprehensive Assay Plus; sWGS, shallow whole genome sequencing with *BRCA1/2* classifier score; WGS, whole genome sequencing

All 695 patients with a successful HRD test were also tested successfully for the presence of tumor PV in OC risk genes (Supplementary Table 2). Supplementary Fig. 2B illustrates that 157 out of the 241 patients (65%) had HRD without a PV in one of the HRR genes, whereas 84 (35%) had HRD with a PV in one of these genes, predominantly in *BRCA1* (*n* = 51) or *BRCA2* (*n* = 26), but also in *RAD51C* (*n* = 4), *BRIP1* (*n* = 1), *RAD51D* (*n* = 1), and *BRCA2*/*RAD51C* (*n *= 1). Additionally, 11 cases were found to have a PV in a tumor assessed as non-HRD, of which the details are provided in Supplementary Table 3. Most, 4 out of 6, *PALB2* or *RAD51D* PV were present in this group, and 2 of 5 cases with a *BRCA1/2* PV in this group had a GIS just below the threshold value of 42 of the TSO 500 HRD test (41 and 40). In addition to PV and HRD, the MSI status was reported for 471 of the 695 patients (Supplementary Table 2). MSI was detected in 5 tumors (1%), all of which were in non-HGSOC and non-HRD (Supplementary Table 4).

#### Comparative results of HRD assays

**Fig. 2:**
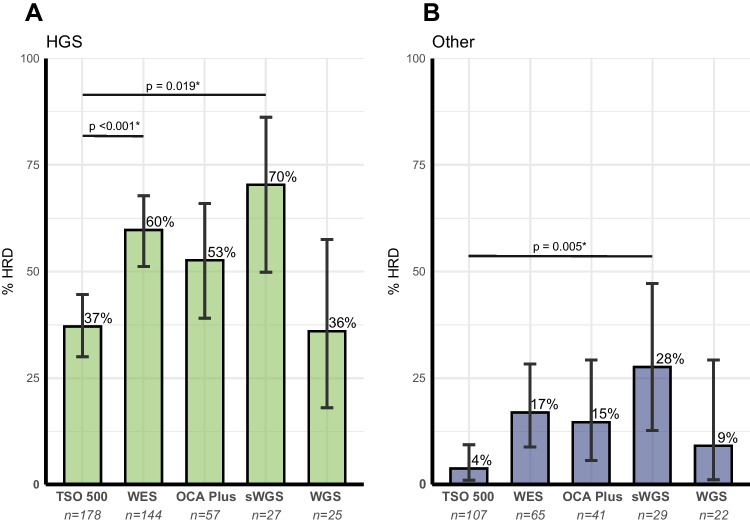
Bar charts representing the percentage of HRD detected using different assays in HGSOC (**A**) and in other histological subtypes (**B**). Statistical differences between the tests were assessed using logistic regression with pairwise comparisons. Asterisk (*) indicates a statistical difference. *P*-values of pairs that were not statistically different are not displayed. Abbreviations: HRD, homologous recombination deficiency; HGS, high-grade serous; HGSOC, high-grade serous ovarian carcinoma; TSO 500, TruSight Oncology 500 HRD test; WES, whole exome sequencing with PureCN algorithm; OCA Plus, Oncomine Comprehensive Assay Plus; sWGS, shallow whole genome sequencing with *BRCA1/2* classifier score; WGS, whole genome sequencing

The total of 765 HRD tests comprised 307 performed with the TSO 500, 241 with WES, 106 with OCA Plus, 62 with sWGS, and 49 with WGS. The test success rates ranged between 87% and 96%. HRD detection rates in successful tests varied among the different test types for both HGSOC and the group of other histological subtypes (Fig. 2). In HGSOC, detection rates ranged from 36% to 70%. Detection was significantly lower with TSO 500 compared to WES (37% compared to 60%, logistic regression, *p*-value < 0.001) and compared to sWGS (37% compared to 70%, logistic regression, *p*-value 0.019). Pairwise comparisons among the remaining test types were not statistically significant. In other histological subtypes, detection rates ranged from 4% to 28%.

**Fig. 3:**
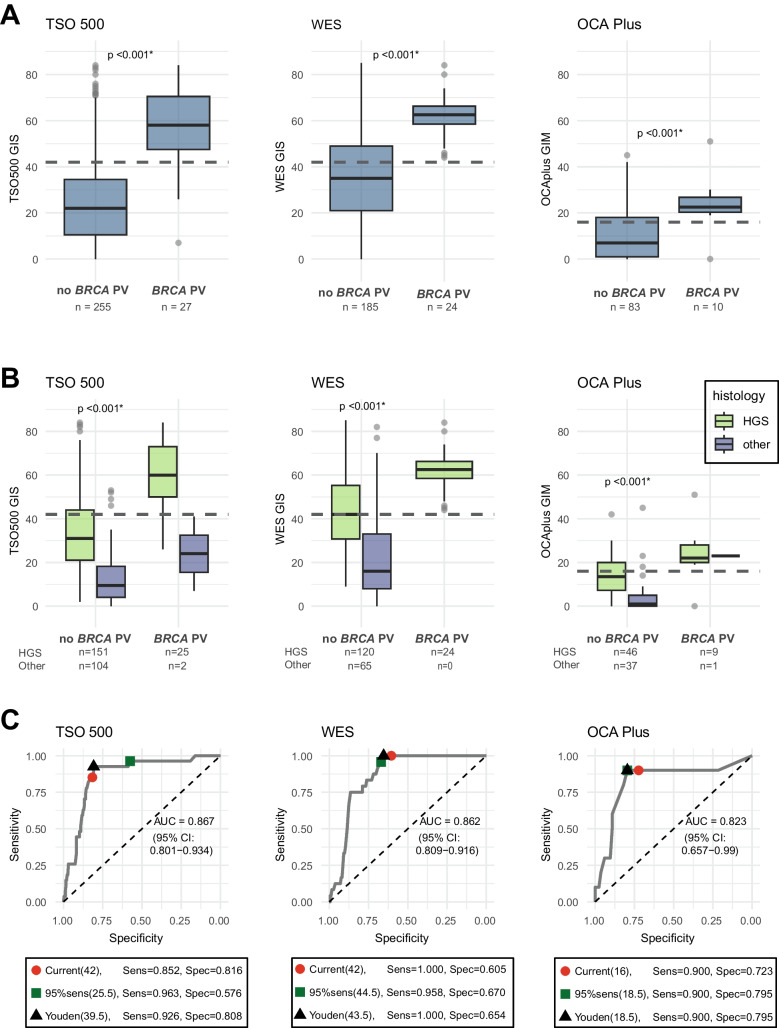
**A **Boxplots of GIS/GIM scores of tumors with and without a *BRCA1/2* PV, for each assay: TSO500, WES, and OCA Plus. Statistical difference was assessed using a Mann–Whitney *U* test. Asterisk (*) indicates statistical difference **B** Boxplots for each assay of GIS/GIM scores of tumors with and without a *BRCA1/2* PV, stratified by HGSOC and other histological subtypes. Statistical difference in the *BRCA1/2*-negative cases was assessed using a Mann–Whitney *U* test. Asterisk (*) indicates statistical difference. Statistical testing was not performed for cases with a *BRCA1/2* PV due to the small sample size **C** ROC curves for each assay evaluating their performance in detecting *BRCA1/2* PV. The current threshold and two optimal thresholds are visualized: one that is closest to 95% sensitivity, and the Youden optimum, which maximizes both sensitivity and specificity Notes. (i) Samples with HRD status but missing scores were not included in the figures (OCA Plus: *n* = 5, TSO 500:* n* = 3). (ii) These graphs could not be generated for the other two assays due to the nature of the *BRCA1/2* classifier score, and the incomplete reporting of the CHORD scores. Abbreviations: TSO 500, TruSight Oncology 500 HRD test; WES, whole exome sequencing with PureCN algorithm; OCA Plus, Oncomine Comprehensive Assay Plus; HGS, high-grade serous; AUC, area under the curve; Sens, sensitivity; spec, specificity; ROC, receiver operating characteristic

 The GIS from the TSO 500, WES and the GIM from OCA Plus were significantly higher for the tumors with a *BRCA1/2* PV compared to the tumors without a *BRCA1/2* PV (Mann Whitney U test, *p* < 0.001 for each assay), as presented in Fig. 3A. Figure 3B shows that, in the tumors without a *BRCA1/2* PV, the scores were significantly higher for the HGSOC compared to other histological subtypes for each assay (Mann Whitney U test, *p *< 0.001 for each assay). Although *BRCA1/2* PV detection is not the aim of the HRD testing, ROC-curves (presented in Fig.  3C) were made to enable comparison of the three assays. Overall, the three tests have similar performance in *BRCA1/2* detection with AUC of 0.87 (95% 0.80–0.93), 0.86 (95% CI 0.81–0.92) and 0.82 (95% CI 0.66–0.99), for the TSO 500, WES and OCA Plus, respectively. The sensitivity to detect a *BRCA1/2* PV of the three tests at the current threshold did not differ significantly (Fisher’s exact test, *p* = 0.133), but the specificity did vary significantly (χ^2^ test, *p* < 0.001), being highest for the TSO 500 (0.816) and lowest for WES (0.605). Based on Youden’s index, the optimal threshold for *BRCA1/2* PV detection appeared lower for TSO 500 and higher for WES and OCA Plus in comparison to the current cut-off. The performance of the assays to detect all OC risk PV is shown in supplementary Fig. 3.

## Discussion

This study informs on the reliability and uniformity of HRD testing in routine diagnostics in all eight Dutch national diagnostic centers that perform Tumor–First genetic testing for OC. Interlaboratory quality rounds, promoter hypermethylation testing, and monitoring of diagnostic outcomes indicate that the HRD assays perform adequately and confirm the applicability of genomic instability as a measure for HRD. However, the observed variability among the different assays, both in their performance during the quality rounds and in the proportion of tumors assessed as HRD in routine diagnostics, suggests that the current HRD tests may not be sufficiently reliable in guiding therapy selection. Future optimizations aimed at reducing inter-assay variability are necessary.

Previous research demonstrated a high concordance among different HRD tests, suggesting that many tests may be used in a clinical context [15, 19–22]. However, our study found large inter-assay variation in the percentage of HRD detected in HGSOC in routine diagnostics. This variation, detection rates ranging from 36 to 70%, has a major impact on patients’ PARP inhibitor treatment eligibility. The two most frequently used HRD assays showed a significant difference in HRD detection rates, being 36% for the TSO 500 and 60% for the WES. Like Myriad MyChoice CDx, that demonstrated an HRD detection rate of 42% in HGSOC [23], both tests use a continuous score based on LST, LOH, and TAI, with a cut-off value of 42. However, variations in HRD detection rates might be explained by differences in assay parameters such as genomic regions and single nucleotide polymorphisms covered, and bioinformatic algorithms. Additionally, HRD detection rates may vary due to differences in minimal neoplastic cell percentages deemed acceptable for HRD evaluation or to suboptimal cut-off values. The ROC curves indicate that adjusting the thresholds for these assays, lowering for the TSO 500 and raising for WES, would improve their performance to detect *BRCA1/2* PV. Nevertheless, *BRCA1/2* PV detection is not the aim of the HRD testing, so while our data suggests threshold optimizations would improve harmonization of HRD testing, additional research is necessary to evaluate this and to assess their clinical utility to predict vulnerability for PARP inhibitors.

The added value of HRD testing lies predominantly in identifying those patients with HRD tumors without a *BRCA1/2* PV that would benefit from PARP inhibitor treatment. This group includes the tumors with *BRCA1* or *RAD51C* promoter methylation. Our study demonstrated that tumors with promoter methylation exhibit high genomic instability scores, confirming the adequate performance of the HRD assays. In tumors assessed as HRD, the genomic instability scores of the tumors with methylation were higher compared to those without. It remains questionable whether those groups differ in their response to PARP inhibitor treatment. A recent single-center study found a better treatment response in the *BRCA1* promoter hypermethylated group compared to the non-HRD group, but no difference compared to the unmethylated HRD-positive group [24]. However, the major PARP inhibitor clinical trials did not include promoter methylation status and thereby it is currently not a biomarker for treatment eligibility.

The quality rounds in our study demonstrated more consistent results in tumors with a PV in or promoter methylation of *BRCA1* or *RAD51C*. This pattern was similar to the results of Andrews et al. who found that positive agreement amongst assays increased when patients with *BRCA1/2* PV were analyzed [21]. Focusing on the inconsistent results, particularly, sWGS showed a notable number of deviations. Together with the observed high frequency of HRD assessed by sWGS in routine diagnostics, this suggests that the *BRCA1/2* classifier is a suboptimal parameter for defining HRD. Furthermore, variation was caused by scores that were close to the threshold. The level of disagreement among tests that can be considered acceptable is uncertain. With a fixed cut-off score delineating HRD from non-HRD, even minor variation can have major clinical consequences, which was also observed in a study comparing the Myriad MyChoice CDx test in a central versus local laboratory [23]. Variation in diagnostic testing is inevitable and with a continuous score that is discretized, perfect consistency among assays is impossible. Nevertheless, when considered alongside HRD detection rates in routine diagnostics, our results highlight the potential for improved uniformity.

Our findings indicate that overall 35% of tumors of a real-world patient cohort were assessed as HRD (49% in HGSOC and 12% in other histological subtypes). This detection rate is lower than the percentage of HRD present in clinical trials such as the PAOLA-1 and PRIMA trial [3, 12], which is likely to be explained by the more restrictive inclusion criteria of those studies. Two major studies assessing HRD detection with Myriad MyChoice CDx reported approximately 40% prevalence of HRD in unselected cohorts [23, 25], though with a higher representation of patients with HGSOC compared to our study. The distribution of HRD with and without a PV in HRR genes observed in our cohort (35% versus 65%) was comparable to those studies [23, 25]. Additionally, similar to Denkert et al. [23], our results highlight that HGSOC more often exhibit genomic instability compared to non-HGSOC. In the Netherlands, all subtypes were tested because HRD testing was incorporated in the Tumor-First workflow, which includes testing all OCs for the presence of tumor PV as a pre-selection to germline testing [13]. In other settings, it may be more effective to restrict HRD testing to HGSOC, while focusing MSI testing on non-HGS tumors.

The strengths of this study include its nationwide scope, the use of daily diagnostic data, and the combination of multiple quality assessments. A limitation of evaluating daily diagnostic practice is the absence of a “golden standard” test, which prevents us from determining the true presence of false positives or false negatives. Although our analyses revealed differences in HRD detection rates, it remains unknown which test achieves the most optimal prediction of response to PARP inhibitors. The Myriad MyChoice CDx test is commonly used as a golden standard in other studies, but is also not a perfect predictor for PARP inhibitor response. Ideally, one would want to investigate the reliability of HRD tests in relation to PARP inhibitor treatment effectiveness. However, these data were not available to us, and conducting such a study would require substantially longer follow-up time. Previous studies, based on the PAOLA-1 trial, have demonstrated that several NGS-based, sWGS, and SNP-based HRD tests are reliable predictors of progression-free survival, with performance comparable to that of the Myriad MyChoice CDx test [26–31]. However, evidence is lacking for the specific HRD tests currently used in the Netherlands, highlighting the need for further research investigating the HRD test that best predicts response to PARP inhibitors.

HRD testing for OC plays an important role in clinical decision-making in many countries, and performing quality control of the implemented tests is essential. This study confirms the applicability of genomic instability as a measure for HRD, but also identifies variation among the different assays. Testing sites should continue to monitor assay performances and to improve consistency and reliability across platforms by performing head-to-head comparisons on the same tumor samples to optimize algorithms and/or thresholds. Harmonizing testing outcomes is important to enhance the reliability of HRD testing for determining PARP inhibitor treatment eligibility.

## Supplementary Information

Below is the link to the electronic supplementary material.

ESM 1 (6.07 MB PDF)

## Data Availability

The datasets generated during and/or analyzed during the current study are available from the corresponding author on reasonable request.
