# Supplementary material for: Evaluation of homologous recombination testing in ovarian carcinoma
Source: Virchows Arch. 2026 Feb 7;488(4):777–88. doi: 10.1007/s00428-026-04432-2 (PMC13053547; doi:10.1007/s00428-026-04432-2)
Supplement: Supplementary file 1 — (6.07 MB PDF) [file 428_2026_4432_MOESM1_ESM.pdf]

Supplementary Information

Evaluation of homologous recombination testing in ovarian carcinoma

Vera M. Witjes, Joanne A. de Hullu, Angela van Remortele, Lilian Vreede, Efraim H. Rosenberg, Saskia A.G.M. Cillessen, Floris H. Groenendijk, Elisabeth M.P. Steeghs, Laura Moonen, Arjen R. Mensenkamp, Arja ter Elst, Wendy W.J. de Leng, Nicoline Hoogerbrugge, Marjolijn J.L. Ligtenberg

*Corresponding Author: Marjolijn J.L. Ligtenberg,

Department of Pathology, Radboud university medical center, Nijmegen, the Netherlands

Department of Human Genetics, Radboud university medical center, Nijmegen, the Netherlands

Marjolijn.Ligtenberg@radboudumc.nl

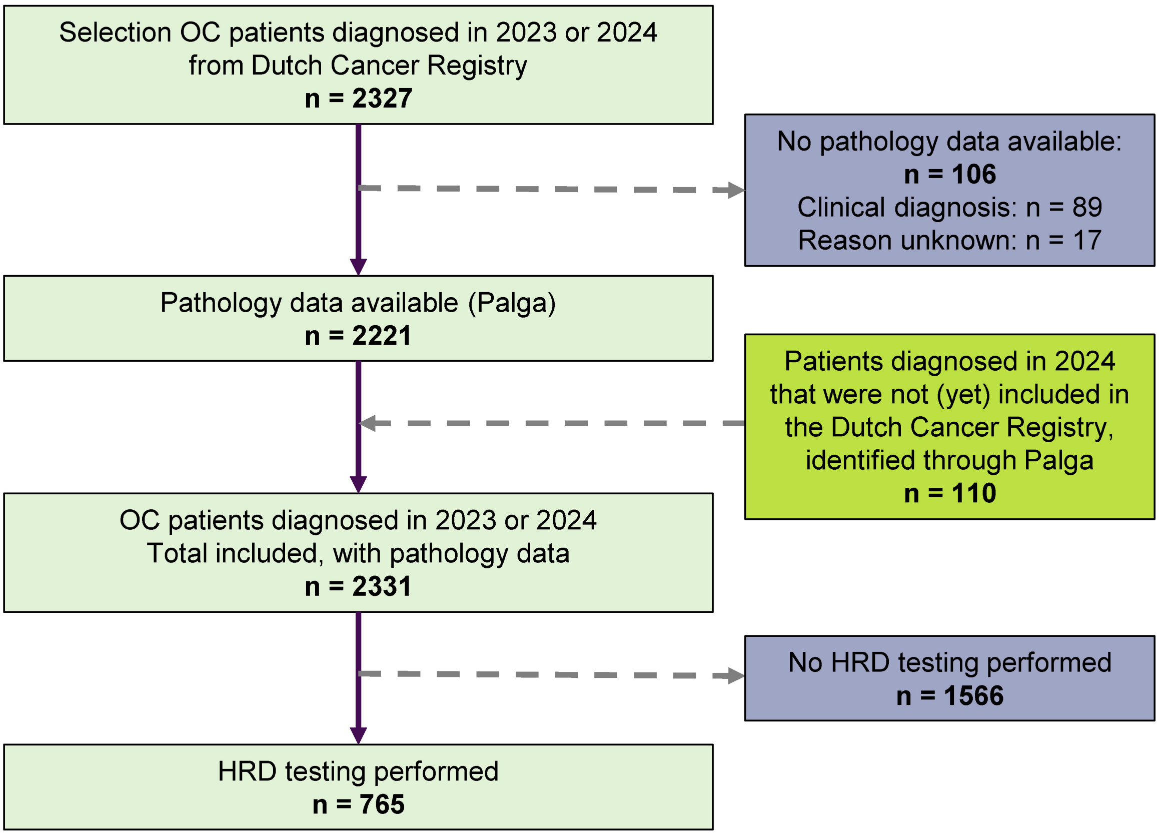

Supplementary Figure 1. Selection route of patients diagnosed in 2023 or 2024 for which HRD testing was performed.

Abbreviations: OC = ovarian carcinoma, HRD = homologous recombination deficiency

Supplementary Table 1. Histological subtypes of the HRD tested population compared to the total OC population for which pathology reports were available.

**Histology**	**HRD tested**	**Total population**	P-value*
High-grade serous	465 (60.8%)	1313 (56.3%)	**<0.001**
Clear cell	57 (7.5%)	155 (6.6%)	
Mucinous	52 (6.8%)	180 (7.7%)	
Endometrioid	45 (5.9%)	153 (6.6%)	
Low-grade serous	45 (5.9%)	114 (4.9%)	
(Adeno)carcinoma, NOS	39 (5.1%)	232 (10.0%)	
Serous, NOS	31 (4.1%)	110 (4.7%)	
Carcinosarcoma	21 (2.7%)	42 (1.8%)	
Other	10 (1.3%)	32 (1.4%)	
**Total**	765 (100.0%)	2331 (100.0%)	

** Chi-square test*

Abbreviations: HRD = homologous recombination deficiency, OC = ovarian carcinoma, NOS = not otherwise specified

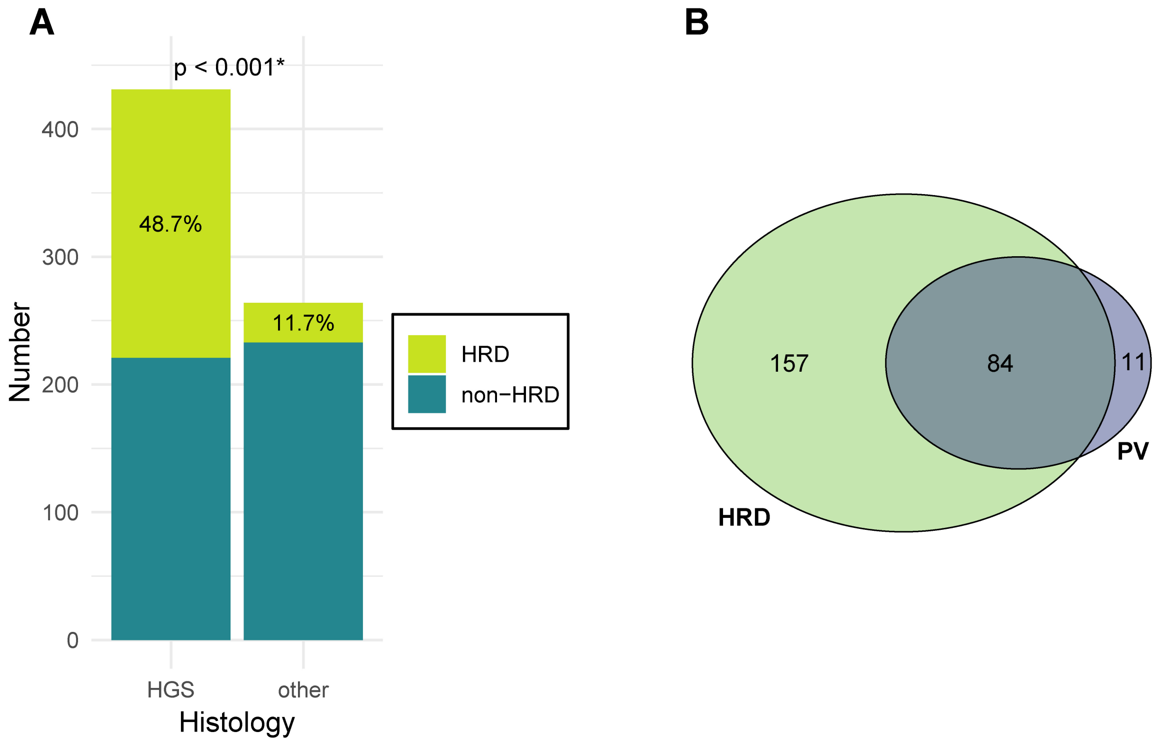

Supplementary Figure 2.
A. Stacked bar chart showing the presence of HRD across HGSOC and other histological subtypes. Statistical differences were evaluated using a Chi-square test. *indicates a significant difference.
B. Co-occurrence of the detection of HRD and tumor PV in an homologous recombination repair gene associated with ovarian cancer depicted using a Venn diagram.

Abbreviations: HGS = high-grade serous, HGSOC = high-grade serous ovarian carcinoma, HRD = homologous recombination deficient, PV = pathogenic variant.

Supplementary Table 2. Characteristics of the Tumor-First PV-tests and MSI tests performed among OC patients that received HRD testing.

**Tumor-First PV tested** (N=695)	Yes	695 (100.0%)
	No	0 (0.0%)
**Tumor-First successful** (N=695)	Yes	695 (100.0%)
	No	0 (0.0%)
**Tumor-First test result** (N=695)	No PV identified	600 (86.3%)
	PV identified	95 (13.7%)
**PV gene** (N=95)	*BRCA1*	55 (57.9%)
	BRCA2	27 (28.4%)
	RAD51C	5 (5.3%)
	PALB2	3 (3.2%)
	RAD51D	3 (3.2%)
	BRIP1	1 (1.1%)
	BRCA2 & RAD51C	1 (1.1%)
**MSI tested** (N=695)	Yes	489 (70.4%)
	No	206 (29.6%)
**MSI test successful** (N=489)	Yes	471 (96.3%)
	No	18 (3.7%)
**MSI test result** (N=471)	No MSI	458 (97.2%)
	Inconclusive	8 (1.7%)
	MSI	5 (1.1%)

Abbreviations: PV = pathogenic variant, MSI = microsatellite instability, OC = ovarian carcinoma, HRD = homologous recombination deficiency

Supplementary Table 3. Detailed information on the non-HRD tumors with a PV in an ovarian cancer risk gene.

PV gene	Nomenclature	Variant	VAF (%)	TC (%)[1]	TC (%)[2]	Histology	Assay	HRD Score
BRCA1	NM_007294.4	c.3624_3625del p.Lys208fs	70	60	60	Serous, NOS	TSO500	GIS: 41
BRCA1	NM_007294.4	c.302-1G>A p.?	16	20	14	HGS	TSO500	GIS: 40
BRCA1	NM_007294.4	c.5333-1G>T p.?	10	n.s.	n.s.	HGS	TSO500	GIS: 26
BRCA1	NM_007294.4	c.1287dup p.Asp430fs	52	60	n.s.	Mucinous	TSO500	GIS: 7
BRCA2	NM_000059.3	c.5213_5216del p.Thr1738fs	57	30	n.s.	HGS	OCA Plus	GIM: 0
PALB2	NM_024675.4	c.3426_3429del p.Leu1142fs	83	80	78	LGS	OCA Plus	GIM: 3
PALB2	NM_024675.4	Deletion of part of exon 5 up to and including exon 13	n.s.	n.s.	16	HGS	WGS	CHORD: n.s.
PALB2	NM_024675.4	c.109-17_131del p.?	n.s.	n.s.	n.s.	HGS	TSO 500	GIS: 32
RAD51C	NM_058216.3	c.224dup p.Tyr75*	58	65	n.s.	HGS	TSO 500	GIS: 38
RAD51D	NM_002878.4	c.141C>G p.Tyr47*	76	80	n.s.	HGS	sWGS	B1/B2 cs: 0/0.17
RAD51D	NM_002878.4	c.144+1G>C p.?	15	60	77	HGS	TSO 500	GIS: 25

[1]*Tumor cel percentage estimated by pathologist*

[2]*Tumor cel percentage calculated via bioinformatic pipeline.*

Abbreviations: HRD = homologous recombination deficiency, PV = pathogenic variant, VAF = variant allele frequency, TC = tumor cel percentage, n.s. = not specified, HGS = high-grade serous, LGS = low-grade serous, NOS = not otherwise specified, TSO 500 = TruSight Oncology 500 HRD test, OCA Plus = Oncomine Comprehensive Assay Plus, sWGS = shallow whole genome sequencing with BRCA1/2 classifier score, WGS = whole genome sequencing, GIS = genomic instability score, GIM = genomic instability metric, B1/B2 cs = BRCA1/BRCA2 classifier score, CHORD = Classifier of homologous recombination deficiency

Supplementary Table 4. Histological subtypes of tumors with HRD, with and without a PV in an ovarian cancer risk gene, and MSI.

Histology	HRD, with PV<br>N = 84<br>n (%)	HRD, without PV<br>N = 155<br>n (%)	MSI<br>N = 5<br>n (%)
High-grade serous	74 (88.1%)	136 (86.6%)	0 (0.0%)
Endometrioid	3 (3.6%)	1 (0.6%)	3 (60%)
Clear cell	0 (0.0%)	2 (1.3%)	0 (0.0%)
Mucinous	0 (0.0%)	2 (1.3%)	1 (20%)
Low-grade serous	1 (1.2%)	0 (0.0%)	0 (0.0%)
Carcinosarcoma	1 (1.2%)	4 (2.5%)	0 (0.0%)
Other	1 (1.2%)	0 (0.0%)	1 (20%)
Serous, NOS	2 (2.4%)	3 (1.9%)	0 (0.0%)
(Adeno)carcinoma, NOS	2 (2.4%)	9 (5.7%)	0 (0.0%)

Note: Histologic subtypes reflect the original diagnostic evaluation and were not reviewed by a single gynecologic pathologist.

Abbreviations: HRD = homologous recombination deficiency, PV = pathogenic variant, MSI = microsatellite instability, NOS = not otherwise specified

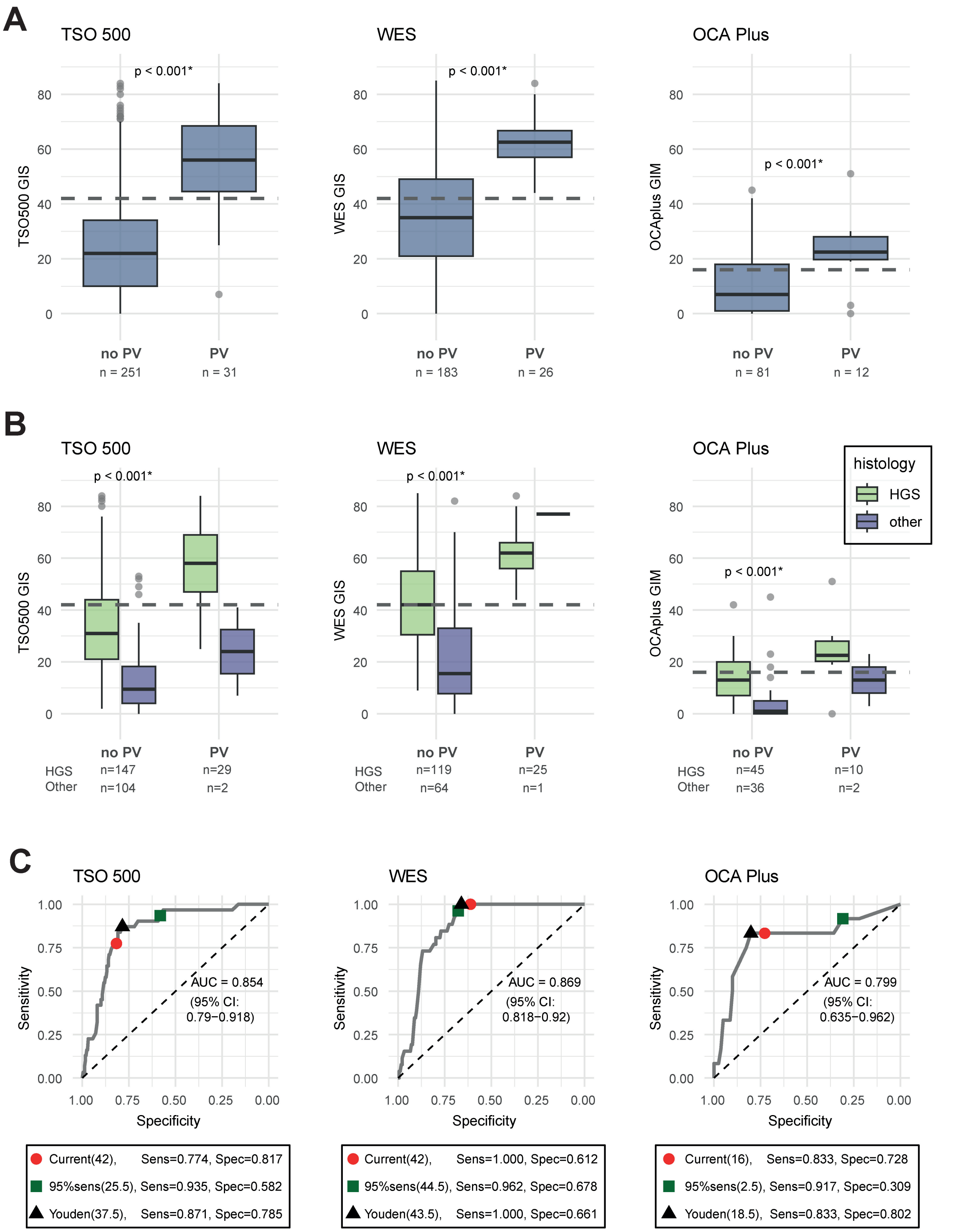

Supplementary Figure 3.

A. Boxplots of GIS/GIM scores of tumors with and without a PV in an OC risk gene, for each assay: TSO500, WES and OCA Plus. Statistical difference was assessed using a Mann-Whitney U test. *indicates statistical difference.

B. Boxplots for each assay of GIS/GIM scores of tumors with and without a PV in an OC risk gene, stratified by HGSOC and other histological subtypes. Statistical difference in the PV-negative cases was assessed using a Mann-Whitney U test. *indicates statistical difference. Statistical testing was not performed for cases with a PV due to the small sample size.
C. ROC curves for each assay evaluating their performance in detecting PV in OC risk genes. The current threshold and two optimal thresholds are visualized: one that is closest to 95% sensitivity, and the Youden optimum, which maximizes both sensitivity and specificity.

Notes. (i) Samples with HRD status but missing scores were not included in the figures (OCA Plus: n=5, TSO 500: n = 3). (ii) These graphs could not be generated for the other two assays due to the nature of the BRCA1/2 classifier score, and the incomplete reporting of the CHORD scores.
Abbreviations: TSO 500 = TruSight Oncology 500 HRD test, WES = whole exome sequencing with PureCN algorithm, OCA Plus = Oncomine Comprehensive Assay Plus, HGS = high-grade serous, AUC = area under the curve, Sens = sensitivity, spec = specificity, ROC = receiver operating characteristic.
